# Determinants of Disability in Minority Populations in Spain: A Nationwide Study

**DOI:** 10.3390/ijerph18073537

**Published:** 2021-03-29

**Authors:** Javier Casillas-Clot, Pamela Pereyra-Zamora, Andreu Nolasco

**Affiliations:** Research Unit for the Analysis of Mortality and Health Statistics, Department of Community Nursing, Preventive Medicine, Public Health and History of Science, University of Alicante, 03080 Alicante, Spain; jcc103@gcloud.ua.es (J.C.-C.); nolasco@ua.es (A.N.)

**Keywords:** disability evaluation, emigrants and immigrants, Roma, ethnicity, inequalities, middle-aged and older adults, health status indicators, social determinants of health, surveys and questionnaires, Spain

## Abstract

Some population groups could be especially vulnerable to the effects of population ageing. The Global Activity Limitation Indicator (GALI) has been proposed as a measure of disability, but it has not been used in minority groups. The aim of this study is to estimate the prevalence of disability using the GALI and to analyse its determinants in immigrant and Roma populations. Data from the Spanish National Health Survey 2017 and the National Health Survey of the Roma Population 2014 were used, including adults aged 50 and above. Prevalence of disability was estimated, and odds ratios were calculated using logistic regression models to assess the association between disability and demographic, socioeconomic, and health variables. The prevalence of disability was estimated at 39.4%, 30.6%, and 58.7% in the native, immigrant, and Roma populations, respectively. Gender was a common determinant for the native and Roma populations. On the other hand, among immigrants, the risk of disability increased over the time residing in Spain. There were significant interactions with age and gender in the native population. Disability has different determinants in the three population groups. Public health measures to protect the Roma population and immigrants’ health should be considered.

## 1. Introduction

Population ageing and life expectancy increase have led to a rise in disability and long-term illnesses [1]. This process has been occurring along with an increase in health inequalities, since there is a close relationship between disability and poverty and lack of resources [2].

The World Health Organisation (WHO) considers social exclusion as one of the main causes of health inequalities [3]. Moreover, some studies suggest that racial and ethnic segregation, as well as different forms of discrimination, may negatively impact the health and disability status of some groups [4,5]. Socially excluded people suffer from deprivation and lack of resources, which may affect health. However, a holistic view should be taken on Social Determinants of Health (SDH), considering conditions of daily life and social structures [3]. In addition, although the SDH approach focusses on indirect causes of health problems, such as ethnicity and migrant status, it has proved to be essential for improving health equity [6,7]. In Europe, minority groups, such as Roma and immigrant populations, are among the most socially vulnerable groups and have less access to the health system, which could lead to a worse health and disability status [8,9].

In 2017, the immigrants residing in Spain represented 13.3% of the population. Of that percentage, 38.3% came from South and Central America, 18.2% came from Africa, and 7.2% from Asia, whereas 34.5% came from other European countries (29.1% from the EU-28 and 5.4% from the rest of Europe), and the remaining came from North America and the Pacific. The most representative, by country of birth, were Moroccans, Rumanians, and Ecuadorians. [10]. Disability and functional limitations prevalence in this population group has been barely studied, despite evidence that they are socially and occupationally vulnerable groups with a greater risk of social exclusion [11,12]. It is also known that immigrants in Spain are more likely to accept jobs with a higher health risk [13] and that they have less access to the health system [14]. On the other hand, some studies conducted in countries with a long immigration history, such as the United States and Canada, have described better health in immigrants than in the general population. This “paradox” has been called the healthy migrant effect [15,16], and it has also been observed and largely discussed in several countries [17,18,19,20]. It has also been noted that immigrants’ health tends to deteriorate over the time residing in the host country, even in the following generations [21,22,23]. Acculturation and assimilation into the cultural mainstream of the host society (such as dietary habits) could be determinant in immigrants’ health deterioration [24,25]. While some studies seem to support this effect in Spain [26,27], contradictory results have been found [28].

As for the Roma population with Spanish nationality, it is estimated that between 700,000 and 970,000 could be living in Spain (between 1.2% and 2.1% of the Spanish population), which renders them one of the largest ethnic minorities [29,30]. Ancient nomads from Punjab settled in Spain more than 500 years ago, and since then, they have been systematically persecuted. It was not until the arrival of democracy in Spain in 1978 that Roma were no longer discriminated against systematically by the institutions [31]. However, although they enjoy full citizenship, they are still one of the most discriminated against and underprivileged groups in the country, with the lowest schooling rates (14.5% of illiteracy and 30.6% of functional illiteracy), very low political and social participation, and mostly belonging to the lowest socioeconomic strata [32,33]. As a consequence, they present worse indicators in the limitations of daily life and most health areas than the most disadvantaged social groups of the general population, presenting health problems at earlier ages, especially among women [34]. Despite the implementation of the National Strategy for Roma Inclusion 2012–2020, no improvement in their health was observed between 2006 and 2014 [35]. In addition, lower use of health services and lower levels of social participation have been observed, with worse perceived health and higher prevalence of obesity, tooth decay, tobacco use (in men), and mental illness [36,37].

Disability is closely related to the social and personal environment, so it might be affected by SDH such as access to self-care, the neighborhood, and the community context [38]. In order to monitor the disability status of the population, the Global Activity Limitation Indicator (GALI) has been proposed [39,40], which has proven to be an effective measure of participation restriction in the general population (although it has not been used to monitor disability in minority groups). It has the advantage of being included in all European health surveys, and it has proven to be valid and reliable in measuring participation restriction (rather than functional limitations, as the name might suggest) [41], and also to be a good predictor of disability [42].

Disability in immigrant and Roma populations has been poorly studied in Spain. Therefore, the aim of this study is to estimate its prevalence using the GALI and to analyse its demographic, socioeconomic, and health status determinants, comparing them with those of the native population (Spanish-born population).

## 2. Materials and Methods

This is a cross-sectional study. Since the prevalence of disability increases with age, particularly in middle-aged adults, data from the population aged 50 years and above were used from the Spanish National Health Survey 2017 (ENSE) [43] and the National Health Survey of the Roma Population 2014 (ENSPG) [37], which were both provided by the Spanish Ministry of Health. The total sample consisted of 10,668 (9879 of the native population and 790 of the immigrant population) participants of the ENSE and 461 participants of the ENSPG.

The result variable considered was the GALI (Disabled/Not Disabled), which is collected through the same question in both health surveys: “For at least the past 6 months, to what extent have you been limited because of a health problem in activities people usually do?” It has three possible answers: Severely limited, limited but not severely, and not limited at all. The first two answers were grouped into a category representing those who were disabled, and the third answer represented those who were not disabled.

The following demographic and socioeconomic variables were considered: sex (Male/Female), age (50 to 64/65+ years), educational level (No studies/Primary/Secondary or University), employment status (Working/Unemployed/Retired/Other situations), and household income (Low/Medium/High). The category “other situations”, in the employment situation variable, included students, people with an incapacity to work, and people who do household work. The household income variable has been collected through slightly different response categories in the two surveys. In the ENSE, it has been grouped by the following categories: less than 1050 euros/from 1050 to 1800 euros/more than 1800 euros per month, and in the ENSPG: less than 950 euros/from 950 to 1950/more than 1950 euros per month, being denominated as low, medium, and high income. For the immigrant population, the time of residence in the country was included in the analyses (Less than 10 years/More than 10 years).

Health variables included were self-rated health status (SRH) (Healthy/Unhealthy), overweight/obesity (Yes/No), and physical (Yes/No) and mental (Yes/No) illnesses. The physical illnesses variable included an affirmative response to at least one of the 12 illnesses on the list in both surveys: high blood pressure, osteoarthritis, chronic allergy, asthma, chronic bronchitis, emphysema, chronic obstructive pulmonary disease (COPD), diabetes, stomach or duodenal ulcer, high cholesterol, migraine, and osteoporosis. Mental illnesses included depression, anxiety, and other mental illnesses.

To describe the demographic, socioeconomic, and health characteristics in all three populations, frequencies and percentages were calculated with their 95% confidence intervals (95% CI). The prevalence of disability and their 95% CIs were also calculated for all three populations. As a measure of association, simple and adjusted Odds Ratios (OR) were calculated using binary logistic regression models, including as explanatory variables the demographic, socioeconomic, and health variables with a significant effect (*p* < 0.05). Due to the complex sample design of the surveys, the weights provided in the surveys were used to produce all the estimations. The statistical programme SPSS v.25^®^ was used for the computations.

## 3. Results

Table 1 shows the characteristics of the native, immigrant, and Roma populations according to demographic, socioeconomic, and health variables. According to sex, a balanced distribution was observed in the native and Roma populations, in contrast to the immigrant population, which shows a higher percentage of women (59.2%). Immigrants were the youngest population group, with an average age of 60.3 years, compared to an average of 65.9 years in the native population and 61.8 years in the Roma population. Immigrants and Roma were the largest population groups in working ages (74.1% and 66.4% respectively), and immigrants had the highest percentage of the working population (43.4% compared to 29.2% in the native population and 25.9% in the Roma). With regard to educational level, the immigrants had the highest proportion of secondary or university studies (73.6% as opposed to 53.1% in the native population), and the percentage was similar among those with no studies (3.2% as opposed to 2.9%). However, among the Roma, the percentage of those without studies reached 27.3%, and only 1.8% had secondary or university studies. The percentage of the Roma with low incomes (83.8%) was much higher than the rest.

Table 2 shows the prevalence of limitations in native, immigrant, and Roma populations. On analysing the difference between immigrants and natives, it can be observed that immigrants had a lower prevalence of limitations than natives (30.6% vs. 39.4%). In the case of the Roma population, this prevalence reached 58.7%. By sex, it can be observed that women had a higher prevalence of limitations than men, particularly in natives (44.5% vs. 33.8%). Likewise, Roma women had a higher prevalence of limitations than men (68.8% vs. 47.9%), although among immigrants, the difference was slight (31.0% vs. 29.8%). According to age, a higher prevalence of limitations was noticed in people aged 65 and above, particularly in the native population (51.0%) and in the Roma population (75.5%), while among immigrants, there were scarce differences between age groups (31.9% vs. 30.2%). In overall terms, there is a clear social gradient, both in the native and in the Roma populations. People who were not working had a low educational level or a low household income had a higher prevalence of disability. In the case of immigrants, there is no clear gradient, with a higher prevalence of disability in unemployed people (32.6%), in other situations (47.9%), in those with primary education (41.5%), and in people with medium incomes (39.0%). It was also observed that immigrants who had been residing in Spain for 10 years or more had a greater prevalence of disability (32.7% vs. 18.9%).

Describing the prevalence according to health variables, it was higher among those who had health problems in all three populations: those who had overweight/obesity (40.5% natives, 36.8% immigrants, and 55.3% among Roma), suffered from physical illnesses (46.1%, 38.7%, and 64.2% respectively), and especially those who suffered from mental health problems (65.0%, 61.2%, and 88.3% respectively). According to self-rated health, the prevalence of disability in those who had a bad perception of their health was 74.5% among Roma, while in natives, it was 69.5% and in immigrants, it was 60.0% (see Table 2).

Table 3 and Table 4 show the association between disability and demographic, socioeconomic, and health variables in all three populations. In the native population, a statistically significant adjusted association was observed between disability and the demographic, socioeconomic (except for household income), and health variables (except for overweight). Women show a higher disability risk than men, and people aged over 65 show a higher risk than the younger population. In addition, a clear risk gradient was also noticed among people who were not working. In particular, a higher risk was observed among people who were in other situations or were retired. People with a lower level of education were at more risk than those with a high educational level. Similarly, people who suffered from physical or mental illnesses and especially those who had a bad perception of their health had a greater risk of disability. By excluding self-rated health from the model, it is shown that sex stopped being significantly associated (*p* = 0.052), but the value of the Odds Ratios in the physical and mental illness variables increased. By excluding self-rated health from the model, it is shown that sex stopped being significantly associated (*p* = 0.052), but the value of the Odds Ratios in the physical and mental illness variables increased.

In the case of the immigrant population, no demographic (except years of residence) or socioeconomic variable was significant. Immigrants who resided in Spain for 10 years or more had a greater risk of disability than those who resided less time in Spain. Oppositely, all the health variables were significant, and persons with overweight/obesity presented a greater risk of disability. Moreover, people who suffered from physical illnesses, mental illnesses, and especially those who had a bad perception of their health, had a greater risk of disability. As in the native population, by not including self-rated health, the risk of disability increased among people who were overweight/obese and physically and mentally ill.

In the Roma population, women were at greater risk of disability than men. With regard to the socioeconomic variables, the only significant variable was the employment situation, with retirement being a protective factor and other work situations (housework, inability to work, study, or others) being a risk factor. Among the health variables, those suffering from mental and physical illnesses and those with poor self-rated health had a greater risk of disability. By excluding self-rated health from the adjusted model, a slight increase in the risk of disability in women is noticed. People aged 65 and above were also at greater risk of disability than the younger group. In addition, the risk of disability increased in people who were physically and mentally ill.

As a result of the findings regarding sex, the interactions between this variable and the rest of the explanatory variables in the three populations were tested. A significant interaction between age and sex was only found in the native population, and it was observed that women presented a greater risk of disability (OR = 1.85 (1.58–2.15)) than men (OR = 1.42 (1.21–1.67)) in the older age group. Complementarily, the differences in the risk of disability between men and women were analysed disaggregating by the age groups in the most advanced ages: 65 to 74 years, 75 to 84 years, and over 85 years. It was shown that the risk of disability increased with age in both men and women, although the risk of limitations in women increased to a greater extent in the older age groups. In the immigrant population, no significant interaction between the explanatory variables and sex was found.

Although in the Roma population, the interaction was not significant, it was noticed that the association between age groups and disability varies in men and women in a similar pattern as in the native population. Women aged 65 and above had a higher risk of disability (OR = 2.13 (0.77–5.83)) than men of the same age group (OR = 1.45 (0.62–3.44)). On the other hand, it was observed that retirement was only a protective factor for women, while for men, it was not significant.

## 4. Discussion

This study aimed to analyse the determinants of disability in minority groups in Spain (immigrant and Roma populations) and in the native population. The disability measure used was the GALl, which is a subjective indicator of participation restriction, meaning that it relies on the individual and social perception of illness and disability. Disabled people are discriminated against in a unique form, having less access to healthcare, employment, and education. In this sense, disability can be considered a SDH, leading to worse health outcomes, for example in mental health and obesity [3,38]. When interacting with other SDH such as ethnicity, migrant status, or gender, particular types of discrimination could be observed [7].

This is the first study in Spain that compares those three population groups for the analysis of disability. In the three population groups studied, objective health problems, such as physical or mental illness, as well as the poor perception of health, were found to be a disability risk factor. In addition, important differences were observed in the prevalence of disability, with the Roma population being the most affected and the immigrant population having an even lower prevalence than the native one. However, the results suggest that there are differences in the determinants of disability between the three populations in Spain. A clear social gradient was observed in the native population (more risk of disability according to sex, educational level, employment situation, and income level), which was not observed in immigrants and Roma when adjusting for other variables.

As a social construction, gender is a crosscutting determinant, leading to worse health outcomes and often interacting with other social determinants, such as ethnicity [44]. One aspect to emphasise in the results is the fact that women had a greater risk of disability than men in native and Roma populations, but not in the immigrant population. According to a study by Padkapayeva et al., the differences in work activity limitations between men and women could be fully explained by the mediation of chronic diseases and the type of occupation [45]. In addition, a report from the Canadian National Survey indicated that more than half of women with activity limitations needed help with household work, while only one-third of men needed it [46]. This may suggest that the types of occupations and the greater burden of household work may lead to a greater risk of limitations for women. Indeed, the double burden of housekeeping and employed work out of the house, in addition to violence and discrimination against women, might mediate in gender health inequities [3]. In addition, immigrant women from Western and Northern European countries may have unknown protective factors, since populations from those countries have shown a non-significant association between sex and functional limitations [47]. A large proportion of immigrants over 50 from those countries could explain that there is no difference by sex among immigrants. Finally, the greater impact of ageing on women’s risk of suffering limitations has also been reported in the United States in several racial groups [48].

In the case of the immigrant population, disability was not associated with their economic or educational level. This contradicts the hypothesis that the existence of the healthy migrant effect in Spain is due to their socioeconomic characteristics (higher education level compared with immigrant populations in other European countries) [26,28]. This could indicate that in this group, there are different social patterns that make a different perception of disability, especially considering the great cultural diversity that exists in this population group. A plausible explanation could be that those who migrate to work in other countries are those who have better health and no limitations, especially among those with the least qualified and lowest income jobs, due to the difficulties and risks of migration [19,20]. On the other hand, people from wealthy countries residing in preferred retirement areas in Spain have shown better health outcomes than natives, while immigrants from those countries residing in the rest of Spain have shown an unhealthy migrant effect, so community context has shown to be a strong determinant among immigrants [49]. A large proportion of those European retirement migrants among the immigrant population over 50 could explain why there is no increase in disability risk in older and retired people, as immigrants with disabilities are more likely to return to their countries [19,20]. In any case, despite the type of occupation and the mediation of physical and mental problems, newcomer immigrants seem to have some disability protective factors. Acculturation and social behavior assimilation could be determinants in the disabling process [24,25]. New evidence will be needed in order to test these hypotheses.

To the best of the authors’ knowledge, this is the first study that evaluates the GALI in the Roma population, so there is no background with which to compare the results. However, considering the social exclusion they suffer, those results were expected. Evidence shows that Roma people are one of the most discriminated minority groups in Spain, being usually prejudged and having greater difficulties in finding a house or a job [32]. This has led to a tendency to cluster in the same neighborhoods and jobs, with notable social segregation [33]. In these cultural contexts, women often assume very rigid family-caring roles and have difficulties in being cared for [50]. In fact, the gender pattern observed in disability contrasts with other studies describing poorer health in women [34,51]. Furthermore, the results show that the measures of socioeconomic and health inequality used for the native population are insufficient to explain the differences in disability of this ethnic minority. The fact that the Roma are one of the ethnic groups with the worst health results in Spain is also reinforced, which makes public health action a priority in order to make visible the situation of social exclusion suffered by Roma and to promote inclusive policies to avoid multiple discrimination and multi-dimensional exclusion.

These measures should include political action to protect all workers, in particular immigrants and ethnic minorities, and to enforce existing legislation, surveillance and health promotion at workplaces, improvements in occupational healthcare access, and improvements in communication with preventive health workers [52]. It is also necessary to implement social, labour, and health policies in order to integrate the Roma population more effectively. Institutions and civil society should take action to render the health needs visible and to implement measures on different levels, from economic integration policies to the training of health professionals in the specific problems of the Roma community. Similarly, it is also necessary to promote the active participation of the Roma population in their own health. Researchers should also make an effort to improve the instruments to monitor this population, given the limitation in the information to study the Roma community [53].

This study has the inherent limitations of a cross-sectional study, as well as limitations due to data sources. The first data source limitation is due to the fact that the data from both surveys have been collected in different years, which could slightly affect the comparability of the results. In addition, the ENSE does not ask about ethnicity or culture of origin. So, another limitation is that, as said in the methods, the Roma population could be included to some extent in the native population, not being possible to analyse the information of this population group separately. It was also not possible to study the immigrant population according to the culture of origin.

One limitation is due to the ENSPG, since even if it was designed to be comparable with the ENSE, there are considerable differences. It includes a limited list of illnesses, compared to the list of 29 illnesses in the ENSE. In this study, only the physical and mental illnesses that are common in both surveys had to be considered (12 illnesses included in the ENSPG). Furthermore, it is not asked whether these diseases have been confirmed by a doctor, so the prevalence could be overestimated. Then again, the list of mental illnesses only asks about depression or others, not asking about anxiety, so the prevalence may be underestimated.

## 5. Conclusions

In conclusion, exclusion is a strong determinant of disability, in particular in ethnic minorities and immigrants. Groups such as the Roma suffer a higher risk of disability due to multiple marginalising factors. In addition, when interacting with gender, the effects of social exclusion increase. Then, intersectional approaches should be adopted in order to better understand this process. On the other hand, there is a healthy migrant effect in Spain, showing a lower prevalence of disability, independently of the different demographic, socioeconomic, and health variables. Nevertheless, this effect tends to disappear over the time of residence. Given the existing cultural and ethnic diversity and the large number of immigrants who are long-term residents in Spain, it is necessary to consider the findings of this study and to take occupational health and preventive measures so that the immigrants’ health does not deteriorate over time.

The results of this study constitute a new set of evidence that the high levels of disability among the Roma, especially among women, as compared to the rest of the population, are a result of the years of social exclusion through which they have lived.

## Figures and Tables

**Table 1:** Frequencies and percentages of the characteristics of native, immigrant, and Roma populations according to demographic, socioeconomic, and health variables (2014–1017).

Variables		Natives		Immigrants		Roma	
		*n*	%	*n*	%	*n*	%
Sex	Male	4670	47.3	322	40.8	225	48.8
	Female	5209	52.7	468	59.2	236	51.2
Age	50–64	5012	50.7	585	74.1	306	66.4
	65+	4866	49.3	204	25.9	155	33.6
Employment status	Working	2950	29.2	343	43.4	114	25.9
	Unemployed	697	7.1	132	16.8	70	15.9
	Retired	4581	46.4	196	24.9	83	18.9
	Other situations	1651	16.7	116	14.9	173	39.3
Educational level	No studies	288	2.9	25	3.2	124	27.3
	Primary	4341	43.9	183	23.2	322	70.9
	Secondary/University	5250	53.1	581	73.6	8	1.8
Household income	Low	2416	32.7	233	41.6	325	83.8
	Medium	2455	33.2	164	29.3	57	14.7
	High	2525	34.1	163	29.1	6	1.5
Overweight/Obesity	Yes	6222	66.2	478	63.1	282	83.9
	No	3171	33.8	280	36.9	54	16.1
Physical illnesses	Yes	7705	78.0	520	65.9	380	82.4
	No	2174	22.0	269	34.1	81	17.6
Mental illnesses	Yes	2058	20.8	104	13.2	61	13.2
	No	7821	79.2	685	86.8	400	86.8
Self-rated health	Healthy	5459	55.3	484	61,3	150	32.5
	Unhealthy	4420	44.7	306	38.7	311	67.5
Years of residence	<10			123	15.6		
	10+			667	84.4		

**Table 2:** Prevalence of limitations and 95% confidence intervals (95% CIs) in native, immigrant, and Roma populations.

Variables		Global Activity Limitation Index (GALI)								
		Natives			Immigrants			Roma		
		*n*	%	95% CI	*n*	%	95% CI	*n*	%	95% CI
Total	-	3895	39.4	37.9–40.9	241	30.6	24.8–36.4	264	58.7	52.8–64.6
Sex	Male	1578	33.8	31.5–36.1	96	29.8	20.7–38.9	105	47.9	38.3–57.5
	Female	2316	44.5	42.4–46.5	145	31.0	23.5–38.5	159	68.8	61.6–76.0
Age	50–64	1413	28.2	25.9–30.5	177	30.2	23.4–37.0	150	50.2	42.2–58.2
	65+	2482	51.0	49.0–53.0	65	31.9	20.6–43.2	114	75.5	67.6–83.4
Employment status	Working	602	20.4	17.2–23.6	84	24.5	15.3–33.7	50	43.9	30.1–57.7
	Unemployed	181	26.0	19.6–32.4	43	32.6	18.6–46.6	32	47.8	30.5–65.1
	Retired	2248	49.1	47.0–51.2	58	29.6	17.9–41.3	43	53.1	38.2–68.0
	Other situations	865	52.4	49.1–55.7	56	47.9	34.8–61.0	123	73.7	65.9–81.5
Educational level	No studies	225	78.1	72.7–83.5	5	19.2	0.0–53.7	81	66.4	56.1–76.7
	Primary	2136	49.2	47.1–51.3	76	41.5	30.4–52.6	176	56.2	48.9–63.5
	Secondary/ University	1533	29.2	26.9–31.5	161	27.7	20.8–34.6	3	37.5	0.0–92.3
Household income	Low	1210	50.1	47.3–52.9	85	36.3	26.1–46.5	187	58.8	51.7–65.9
	Medium	1020	41.6	38.6–44.6	64	39.0	27.1–50.9	32	59.3	42.3–76.3
	High	746	29.6	26.3–32.9	38	32.2	17.3–47.1	4	66.7	20.5–100.0
Overweight/Obesity	Yes	2516	40.5	38.6–42.4	176	36.8	29.7–43.9	152	55.3	47.4–63.2
	No	1092	34.5	31.7–37.3	56	20.0	9.5–30.5	27	50.0	31.1–68.9
Physical illnesses	Yes	3548	46.1	44.5–47.7	201	38.7	32.0–45.4	239	64.2	58.1–70.3
	No	346	15.9	12.0–19.8	40	14.9	3.9–25.9	25	32.1	13.8–50.4
Mental illnesses	Yes	1341	65.2	62.7–67.7	64	61.5	49.6–73.4	53	88.3	79.6–97.0
	No	2553	32.7	30.9–34.5	178	25.9	19.5–32.3	211	54.1	47.4–60.8
Self-rated health	Healthy	824	15.1	12.7–17.5	58	12.0	3.6–20.4	39	26.4	12.6–40.2
	Unhealthy	3070	69.5	67.9–71.1	183	60.0	52.9–67.1	225	74.5	68.8–80.2
Years of residence	<10	-	-	-	23	18.9	2.9–34.9	-	-	-
	10+	-	-	-	218	32.7	26.5–38.9	-	-	-

**Table 3:** Simple and adjusted odds ratio (OR) and 95% confidence interval (95% CI) according to the explanatory variables in native and immigrant populations.

Variables	Natives						Immigrants					
	Simple Analysis		Analysis Adjusting for All Variables Including SRH ª		Analysis Adjusting for All Variables Not Including SRH ª		Simple Analysis		Analysis Adjusting for All Variables Including SRH ª		Analysis Adjusting for All Variables Not Including SRH ª	
	OR	95% CI	OR	95% CI	OR	95% CI	OR	95% CI	OR	95% CI	OR	95% CI
**Sex**	*		*		NS		*		NS		NS	
Male	1		1				1					
Female	10.57 *	1.45–1.70	1.13 *	1.02–1.26			1.06	0.78–1.45				
**Age**	*		*		*		*		NS		NS	
50–64	1		1		1		1					
65+	2.65 *	2.44–2.88	1.21 *	1.03–1.41	1.25 *	1.06–1.47	1.07	0.76–1.51				
**Employment status**	*		*		*		*				*	
Working	1		1		1		1		NS		1	
Unemployed	1.37 *	1.13–1.66	0.99	0.79–1.25	0.91	0.71–1.16	1.51	0.97–2.34			1.28	0.78–2.08
Retired	3.75 *	3.37–4.17	1.75 *	1.46–2.11	1.76 *	1.45–2.12	1.3	0.88–1.92			1.2	0.76–1.78
Other situations	4.29 *	3.76–4.90	1.89 *	1.59–2.25	2.12 *	1.77–2.54	2.84 *	1.83–4.40			2.15 *	1.32–3.52
**Educational level**	*		*		*		*		NS		NS	
Secondary/ University	1		1		1		1					
Primary	2.35 *	2.16–2.55	1.15 *	1.03–1.29	1.33 *	1.18–1.50	1.84 *	1.30–2.60				
No studies	8.63 *	6.49–11.47	3.33 *	2.38–4.65	3.60 *	2.59–5.00	0.57	0.20–1.60				
**Household income**	*		NS		*		*		NS		NS	
High	1				1		1					
Medium	1.69 *	1.51–1.90			1.19 *	1.05–1.36	2.14 *	1.32–3.45				
Low	2.39 *	2.12–2.68			1.40 *	1.22–1.61	1.91 *	1.22–3.00				
**Overweight/Obesity**	*		NS		NS		*		*		*	
No	1						1		1		1	
Yes	1.29 *	1.18–1.41					2.32 *	1.64–3.28	1.77 *	1.18–2.65	2.19 *	1.51–3.16
**Physical illnesses**	*		*		*		*		*		*	
No	1		1		1		1		1		1	
Yes	4.50 *	3.98–5.09	1.83 *	1.59–2.12	3.10 *	2.64–3.63	3.57 *	2.45–5.21	1.89 *	1.21–2.93	2.46 *	1.65–3.68
**Mental illnesses**	*		*		*		*		*		*	
No	1		1		1		1		1		1	
Yes	3.87 *	3.49–4.29	1.77 *	1.57–2.01	3.10 *	2.74–3.51	4.53 *	2.95–6.97	1.87 *	1.13–3.10	3.23 *	2.03–5.14
**Self-rated health**	*		*				*		*			
Healthy	1		1				1		1			
Unhealthy	12.82 *	11.63–14.14	8.80 *	7.93–9.77			10.95 *	7.66–15.65	8.85 *	6.02–13.01		
**Years of residence**							*		*		*	
<10	-		-		-		1		1		1	
10+	-		-		-		2.06 *	1.28–3.32	1.99 *	1.11–3.56	2.05 *	1.19–3.51

* Statistically significant (*p* < 0.05); NS not significant. ª The final model only included variables with significant adjusted effects.

**Table 4:** Simple and adjusted odds ratio (OR) and 95% confidence interval (95% CI) according to the explanatory variables in the Roma population.

Variables	Roma					
	Simple Analysis		Analysis Adjusting for All Variables Including SRH ª		Analysis Adjusting for All Variables Not Including SRH ª	
	OR	95% CI	OR	95% CI	OR	95% CI
**Sex**	*		*		*	
Male	1		1		1	
Female	2.40 *	1.63–3.52	3.01 *	1.73–5.23	3.10 *	1.86–5.17
**Age**	*		NS		*	
50–64	1				1	
65+	3.06 *	1.98–4.73			2.11 *	1.18–3.78
**Employment status**	*		*		*	
Working	1		1		1	
Unemployed	1.17	0.64–2.14	0.56	0.27–1.16	0.79	0.40–1.56
Retired	1.45	0.82–2.57	0.43 *	0.20–0.94	0.46 *	0.23–0.95
Other situations	3.58 *	2.16–5.93	1.96 *	1.08–3.59	1.56	0.81–2.98
**Educational level**	*		NS		NS	
Secondary/University	1					
Primary	2.14	0.50–9.12				
No studies	3.29	0.75–14.46				
**Household income**	*		NS		NS	
High	1					
Medium	1.02	0.57–1.83				
Low	1.4	0.25–7.76				
**Overweight/Obesity**	*		NS		NS	
No	1					
Yes	1.24	0.69–2.22				
**Physical illnesses**	*		*		*	
No	1		1		1	
Yes	3.81 *	2.26–6.41	2.50 *	1.28–4.88	3.17 *	1.75–5.77
**Mental illnesses**	*		*		*	
No	1		1		1	
Yes	6.42 *	2.85–14.48	4.66 *	1.88–11.51	5.31 *	2.23–12.64
**Self-rated health**	*		*			
Healthy	1		1			
Unhealthy	8.17 *	5.22–12.78	7.58 *	4.53–12.68		

* Statistically significant (*p* < 0.05); NS not significant. ª The final model only included variables with significant adjusted effects.

## Data Availability

The data of the Spanish National Health Survey 2017 can be downloaded from the Spanish Ministry of Health web page https://www.mscbs.gob.es/estadisticas/microdatos.do (accessed 12 February 2020) and the date of the National Health Survey of the Roma Population 2014 can be obtained from the Ministry of Health on demand https://www.mscbs.gob.es/profesionales/saludPublica/prevPromocion/promocion/desigualdadSalud/solicitudFicherosENS.htm (accessed 12 February 2020).
